# The size, burden and cost of disorders of the brain in the UK

**DOI:** 10.1177/0269881113495118

**Published:** 2013-09

**Authors:** Naomi A Fineberg, Peter M Haddad, Lewis Carpenter, Brenda Gannon, Rachel Sharpe, Allan H Young, Eileen Joyce, James Rowe, David Wellsted, David J Nutt, Barbara J Sahakian

**Affiliations:** 1 Hertfordshire Partnership NHS University Foundation Trust, Queen Elizabeth II Hospital., Welwyn Garden City, UK; 2 University of Hertfordshire, Postgraduate Medicine, Hatfield, UK; 3 University of Cambridge, School of Clinical Medicine, Addenbrooke’s Hospital, Cambridge, UK; 4 Greater Manchester West Mental Health NHS Foundation Trust, Cromwell House, Cromwell Road, Eccles, Manchester, M30 0GT; 5 Centre for Lifespan and Chronic Illness Research (CLiCIR), University of Hertfordshire, Hatfield, UK; 6 Leeds Institute for Health Sciences, University of Leeds, Leeds, UK; 7 University of Hertfordshire, Department of Psychology, Hatfield, UK; 8 Division of Brain Sciences, Imperial College, Neuropsychopharmacology, London, UK; 9 Wellcome Department of Imaging, University College London, Institute of Neurology, London, UK; 10 University of Cambridge, Department of Clinical Neurosciences, Addenbrookes Hospital., Cambridge, UK; 11 University of Cambridge, Department of Psychiatry, Addenbrooke’s Hospital, Cambridge, UK; 12 Neuroscience and Psychiatry Unit, University of Manchester, Manchester, UK; 13 British Association for Psychopharmacology Council Member, UK

**Keywords:** Brain disorders, cost, burden

## Abstract

**Aim:**

The aim of this paper is to increase awareness of the prevalence and cost of psychiatric and neurological disorders (brain disorders) in the UK.

**Method:**

UK data for 18 brain disorders were extracted from a systematic review of European epidemiological data and prevalence rates and the costs of each disorder were summarized (2010 values).

**Results:**

There were approximately 45 million cases of brain disorders in the UK, with a cost of €134 billion per annum. The most prevalent were headache, anxiety disorders, sleep disorders, mood disorders and somatoform disorders. However, the five most costly disorders (€ million) were: dementia: €22,164; psychotic disorders: €16,717; mood disorders: €19,238; addiction: €11,719; anxiety disorders: €11,687. Apart from psychosis, these five disorders ranked amongst those with the lowest direct medical expenditure per subject (<€3000). The approximate breakdown of costs was: 50% indirect costs, 25% direct non-medical and 25% direct healthcare costs.

**Discussion:**

The prevalence and cost of UK brain disorders is likely to increase given the ageing population. Translational neurosciences research has the potential to develop more effective treatments but is underfunded. Addressing the clinical and economic challenges posed by brain disorders requires a coordinated effort at an EU and national level to transform the current scientific, healthcare and educational agenda.

## Background

No group of chronic diseases burdens the world more than mental illnesses (World Health Organization (WHO), 2008). Numerous epidemiological studies on mental disorders throughout the world have shown that in each year about one-third of the adult population suffers from a mental disorder (Kessler et al., 2009). Yet, less than one-third receive any form of treatment, suggesting a considerable level of unmet need (Kessler et al., 2009; Wittchen et al., 2011). Neurological disorders, including stroke and dementia, are usually classified separately and involve additional substantial costs.

Mental and neurological illnesses taken together comprise ‘disorders of the brain’ and, according to current estimates, these brain disorders account for approximately 13% of global disease prevalence, surpassing both cardiovascular diseases and cancer (Collins et al., 2011). The total EU healthcare burden of disorders of the brain represents the largest contributor to the ‘all cause morbidity’ burden as measured by disability-adjusted life years (DALYs) and is arguably the number one economic challenge for European healthcare now and for the future (Gustavsson et al., 2011a). Moreover, the Global Burden of Disease studies that cover all disease groups and injury categories (Murray and Lopez, 1997; WHO, 2002, 2008), demonstrate that increasingly higher proportions of the global burden of disease will be attributed to brain disorders over time.

With scarce resources available to invest into health research, difficult choices have to be made on which diseases to prioritize. Cost-of-illness studies can help to inform research priorities by providing estimates of the economic burden of particular health problems (Luengo-Fernandez et al., 2012). According to a 2006 UK governmental review (Cooksey, 2006), the impact of diseases on the population and economy should be used to help determine health research priorities. Therefore, by applying cost-of-illness studies consistently across several diseases, it may be possible to rank diseases according to their economic burden and help plan the allocation of future research funds towards those diseases with the greatest burden (Luengo-Fernandez et al., 2012).

Careful attention to indirect costs is required in any study that attempts to analyse the true burden of individual disorders. Compared with somatic diseases, brain disorders involve disproportionally high indirect costs (i.e. lost production due to work absence or early retirement) and relatively low direct health and social care costs (Collins et al., 2011; Murray and Lopez, 1996; Prince et al., 2007; WHO, 2008; Wittchen and Jacobi, 2005). This means that the application of more effective treatment has the potential to considerably reduce the overall economic burden to society and improve patient quality of life, over and above any reductions in healthcare costs. Thus, there is a clear argument for investing in research that leads to a better understanding of how to most effectively prevent, diagnose, treat and manage brain diseases.

Research into the causes and the treatment of brain disorders and the allocation of appropriate mental health treatment resources and care have been hampered over the years. Impediments include the marginalization and stigmatization of mental illness as well as inter-disciplinary fragmentation of research and practice (Klin and Lemish, 2008; Prince et al., 2007; Wittchen et al., 2011). There is also an impending crisis in drug development (Insel et al., 2012), which requires a major overhaul of translational research into mental health. The worldwide withdrawal of major pharmaceutical companies from key areas of preclinical and clinical neuroscience research (Nutt and Goodwin, 2011) and the trend for industrial research to move away from the UK and Europe, are the more recent and pressing threat to prospects for the advancement of treatment for brain disorders in the UK. It is imperative to redirect and coordinate the UK national research strategy, including governmental policy, industrial partnerships and the third sector contributions. However, to do so successfully requires current and relevant data regarding the whole cost and burden of brain disorders.

Prevalence estimates for health services planning have often been derived from the World Health Organization’s Burden of Disease studies (Murray and Lopez, 1996; WHO, 2002, 2008), and there has been a relative shortage of systematic data on the impairments and disabilities associated with specific brain disorders across the UK. Estimates derived from global data are of relatively limited value for UK research and health planning, as they are heavily influenced by the most populous countries and regions. In addition, collating data from diverse populations with varying disease epidemiology and substantially different socio-economic and healthcare systems obscures country-specific patterns. National and regional specific data are likely to be of more value for planning.

In 2011 the European Brain Council (EBC), together with the European College of Neuropsychopharmacology (ECNP), extended and updated an earlier study, first performed in 2004, estimating the comprehensive cost and burden of all disorders of the brain in Europe (Andlin-Sobocki et al., 2005; Gustavsson et al., 2011a; Wittchen and Jacobi, 2005; Wittchen et al., 2011). Mental and neurological illness data derived from 30 European countries, including the UK, were used to calculate and estimate the patterns and costs of treatment and the health economic implications in terms of total direct and indirect costs for EU nations (Andlin-Sobocki et al., 2005; Wittchen and Jacobi, 2005). Here we present a summary of the epidemiological data for the size, burden and cost of disorders of the brain specifically pertaining to the UK, derived from the EBC/ECNP 2011 studies (Gustavsson et al., 2011a; Wittchen et al., 2011).

Our overarching aim is to strengthen awareness of the impact and cost of disorders of the brain in the UK, including the indirect costs, to focus attention on the scope for improved collaborative research and treatment development across the fields of mental and neurological illness and to identify those areas of most need. We attempt to identify the illnesses that contribute most in terms of global health burden, as well as those for which improvement in available treatment might prove most fruitful in health economic and quality-of life terms, as a guide for future research prioritization within the NHS. Finally, we offer constructive suggestions, based upon some promising recent health initiatives, for example in the field of stroke and dementia, that we believe could make a significant difference in moving the field forward.

## Objectives

The objective of this analysis was to estimate for the UK, in 2010, (1) the number of individuals with disorders of the brain; (2) the total cost per person related to each brain disorder, classified according to direct and indirect costs and (3) the total cost per brain disorder for the UK economy.

## Methods

The EBC/ECNP studies (Gustavsson et al., 2011a; Wittchen et al., 2011) were designed and managed by prominent epidemiologists and health economists. A comprehensive and systematic review of epidemiological data was conducted by panels of European experts; UK summary data were extracted from the European database by members of the EBC team and reviewed by the authors of this paper, including clinicians with expertise in a broad diversity of brain disorders and their epidemiology (NF, PMH, EJ, JBR, DN, AHY) as well as experts in preclinical neuroscience (BJS) statistics (DW, LC) and health economics (BG).

Details of the EBC study methodology have already been published (Gustavsson et al., 2011a; Wittchen et al., 2011). In summary, a stepwise multi-method study approach was adopted consisting of (a) iterative literature searches for epidemiological publications and subsequent data analyses of published material; (b) reanalyses of existing accessible epidemiological data sets; and (c) structured expert inquiries and a questionnaire survey with experts in all EU countries. Only those studies conducted in community samples and reporting prevalence estimates for established diagnoses of mental disorders (according to criteria of DSM-III (APA, 1980), DSM-IIIR (APA, 1987) or DSM-IV (APA, 1994); or ICD-10, (World Health Organization, 1993) or those using at least instruments with explicit diagnostic criteria that allow such inferences, were considered. The results produced consolidated best estimates for the EU total population in the year 2010 for (1) the 12-month prevalence of mental and neurological disorders covering as far as possible all major disorders for children and adolescents (2–17 years), adults (18–65 years), and the elderly (65+ years); (2) the number of persons affected by each diagnosis. The consolidated size and burden data were further used for (3) comprehensive health economic analyses, including cost modelling for all conditions covered. Table 1 details the sources from which all cost information was obtained for the UK.

**Table 1.** Summary of cost data as sourced from Gustavsson et al. (2011a).

Disorder	Author	Year	Data Source
*Anxiety*	Jacobi et al.	2002	Germany
	Priebe et al.	2009	UK
	Priebe et al.	2010	UK
*Addiction*	Balakrishnan et al.	2009	UK
	Godfrey et al.	2004	UK
*Brain tumours*	Blomqvist et al.	1996	Sweden
	Wasserfallen et al.	2005	Switzerland
*Dementia*	Gustavsson et al.	2011	UK
	Wolstenholme et al.	2002	UK
*Epilepsy*	Cockerell et al.	1994	UK
	Jacoby et al.	1998	UK
	Swingler et al.	1994	UK
	Van Hout et al.	1997	UK
*Headache*	Linde et al.	2012	EU
*Mood disorders*	Das and Guest	2002	UK
	Thomas and Morris	2003	UK
*Multiple sclerosis*	Kobelt et al.	2006	UK
*Parkinsons*	McCrone et al.	2007	UK
*Psychosis*	Heider et al.	2009	UK
	Bebbington et al.	2005	UK
*Stroke*	Luengo-Fernandez et al.	2009	UK
*Traumatic brain injury*	Morris et al.	2008	UK
	Polinder et al.	2005	UK
*Child & adolescent disorders*	Knapp et al.	2009	UK
	Hakkaart-van et al.	2007	Holland
	Romeo et al.	2006	UK
Mental retardation*	Polder et al.	2002	Holland
*Eating disorders*	Krauth et al.	2002	Germany
*Personality disorders*	Van Asselt et al.	2007	Holland
	Soeteman et al.	2008	Holland
*Sleep disorders*	Godet-Cayré et al.	2006	France
	Leger et al.	1999	France
	Jennum and Riha	2009	Denmark
	Jennum and Kjellberg	2010	Denmark
	Jennum and Kjellberg	2011	Denmark
*Neuromuscular disorders*	Espérou et al.	2000	France
	Mahdi-Rogers et al.	2009	UK
	AccessEconomics	2007	Australia
	Schepelmann et al.	2010	Germany
*Somatoform disorders*	Jacobi et al.	2002	Germany

* The term “mental retardation” was retained in this paper as this is consistent with the ICD-10 diagnosis. This term is under consideration for a change in the ICD-11.

The total cost of a disorder in the UK was calculated by combining epidemiological (number of patients) and economic data (cost per patient), using a prevalence-based approach which multiplied the total number of UK persons affected by a disorder in a 12-month period (2010), with their mean cost in the same year. There are several approaches to assess the cost of a defined disease. This study considered the costs of all resources used or lost due to the disease, irrespective of who the paying organizations are (employers, families, Department of Work and Pensions, National Health Service, etc.). This perspective is arguably the most relevant for decision makers, whose main interest is the welfare of society as a whole. It is also the relevant perspective for judging if all costs are included (though it is important to include each cost item only once in order to avoid double counting). Costs are presented in three main categories: (a) direct healthcare costs (i.e. all goods and services related to the prevention, diagnosis and treatment of a disorder, for example physician visits, hospitalizations and pharmaceuticals); (b) direct non-medical costs (i.e. other goods and services related to the disorder, for example social services, special accommodation and informal care); and (c) indirect costs (i.e. lost production due to work absence or early retirement). Cost items excluded from the estimates, due to the lack of data or lack of consistent methods on how to value and report these costs, included indirect costs due to premature mortality, intangible costs (i.e. the monetary value of suffering from a disorder), and costs of crime caused by, for example, addiction. Costs related to research were also excluded because they are not considered to be caused by the disorder per se, but rather as an investment aimed at reducing the costs in the future. Moreover, to a large extent, research costs will likely be reflected in the prices of potential treatment interventions coming out of this research.

A ‘bottom-up’ analysis of costs was applied and the study identified as far as possible patient-level data (from UK literature) on annual direct and indirect costs incurred for each disorder (e.g. by ascertaining cost items from interview, questionnaire or review of medical records); the mean cost per person was then multiplied by the estimated number of UK-affected persons to obtain an approximation of the total cost. Adjustments were made to avoid inflated estimates related to the specific sample characteristics. However, in the case that no such data were available for a disorder, estimates from ‘top-down’ national or regional statistics on the total costs of a group of disorders were used. Existing UK-specific data for each disorder were applied to a model that additionally adjusted cost estimates to 2010 values and to the *total UK population with the disorder* where the available treatment-cost estimates only referred to a patient subset, and allowed extrapolation from other countries to fill data gaps (see Gustavsson et al., 2011a for further details).

## Results

Primary ‘patient-level’ UK prevalence data (from literature) was available for the majority of brain disorders. Where the EBC/ECNP data set did not include prevalence data from the UK, data from other EU countries were used; anxiety disorders (Iceland, Norway and Switzerland), psychosis and eating disorder (Germany), stroke (Iceland, Norway and Sweden), headache (Central Europe), brain tumour (Norway and Sweden), somatoform disorder (Central Europe) and some sleep disorders (Norway) and neuromuscular disorders (Central Europe). Table 2 lists the disorders included in the study.

**Table 2.** List of disorders (ICD-10 codes) as sourced from Gustavsson et al. (2011a).

Disorders	ICD-10 codes
Child/adolescent disorders	
Hyperkinetic disorders/ADHD	F90.x
Conduct disorder	F91.x
Pervasive developmental disorders / autism	F84.x
Personality disorders	
Dissocial PD	F60.2
Emotionally unstable PD	F60.3
Dementia	F00–F03
Headache	G44
Mood disorders	
Unipolar/major depression	F32, F33
Bipolar disorders	F30, F31
Neuromuscular disorders	F50.2
Brain tumor	C70–72 D32–33 D42–43
Traumatic brain injury	S06
Psychotic disorders	
Schizophrenia and other psychotic	F2x
disorders and syndromes	
Multiple sclerosis	G35
Addiction	
Alcohol dependence	F10.2
Opioid dependence	F11.2
Cannabis dependence	F12.2
Somatoform disorder Epilepsy	F45
Epilepsy	G40
Parkinson’s disease	G20
Sleep disorders	
Nonorganic insomnia	F51.x
Hypersomnia	G47.1
Narcolepsy	G47.3
Sleep apnea	G47.4
Anxiety disorders	
Panic disorder	F41.0
Agoraphobia	F40.0
Social phobia	F40.1
Generalized anxiety disorder (GAD)	F41.1
Specific phobias	F40.2
Obsessive compulsive disorder (OCD)	F42
Posttraumatic stress disorder (PTSD)	F43.1
Stroke	**161**, 163, 164 167
Mental retardation	F70–F79
Eating disorders	
Anorexia nervosa/atypical AN	F50.0, F50.1
Bulimia nervosa/atypical BN	F50.2, F50.3

### UK prevalence of disorders of the brain

**Figure 1.**
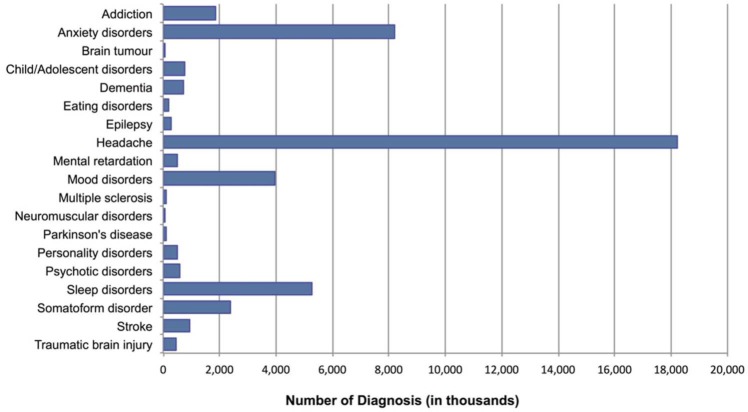
Estimated 12-month UK prevalence of disorders of the brain.

Figure 1 illustrates the estimated 12-month UK prevalence of disorders of the brain included in this study in 2010. The estimated total amounted to roughly 45 million diagnosed cases, ranging between 26,000 cases of brain tumour and 18 million cases of headache. There were greater than 5 million cases of anxiety disorder (8,196, 000 (18.17% of UK population)) and sleep disorder (5,268,0000 (11.68%)) and greater than one million cases of addiction (1,878,000 (4.16%)), mood disorder (i.e. unipolar depression and bipolar disorders) (3,937,000 (8.73%)) and somatoform disorder (2,396,000 (5.31%)).

### UK cost of disorders of the brain

The total UK cost of disorders of the brain in 2010 was estimated at €134,476 million. Indirect costs associated with patients’ productivity losses constituted by far the largest component of the total cost (€62,346 million), comprising 46.4% of the overall cost, whereas the remainder of the cost was divided into 26.8% each for direct non-medical (€36,077 million) and direct healthcare (€36,053 million) costs.

### Cost per subject

**Figure 2.**
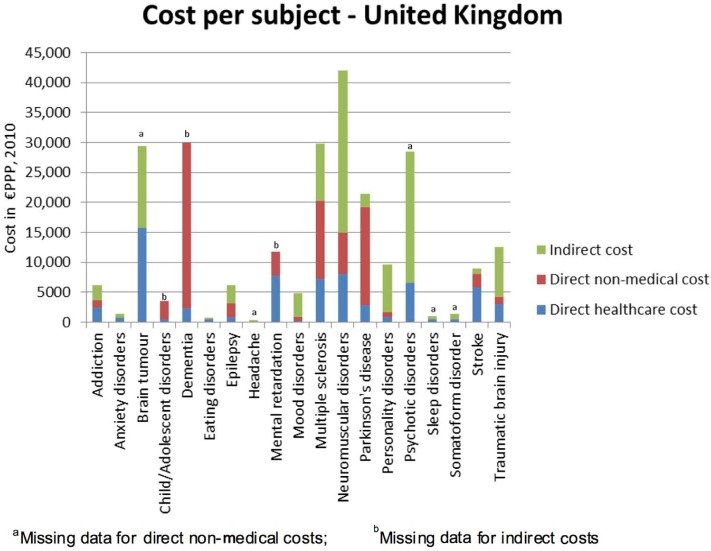
UK per-subject cost of brain disorders.

On average, the estimated UK per-subject cost of brain disorders was €3126, but varied widely according to diagnosis, ranging between €391 for headache and €42,000 for neuromuscular disorders (Figure 2). Disorders for which the per-subject total cost exceeded €20,000 included neuromuscular disorders, brain tumour, dementia, multiple sclerosis, psychosis and Parkinson’s disease. Direct medical expenditure was highest per subject for brain tumour, neuromuscular disorders, mental retardation, multiple sclerosis, psychosis and stroke, whereas the highest direct non-medical costs were associated with dementia, multiple sclerosis and Parkinson’s disease. Neuromuscular disorders, psychosis and brain tumour were also associated with the highest individual indirect costs.

Data on direct non-medical costs for brain tumour, headache, psychotic disorders, sleep disorders and somatoform disorders were missing from the original paper. Data on indirect costs for child/adolescent disorders, dementia and mental retardation were also missing from the original survey. Therefore these were not included in the analysis.

### Total cost

**Figure 3.**
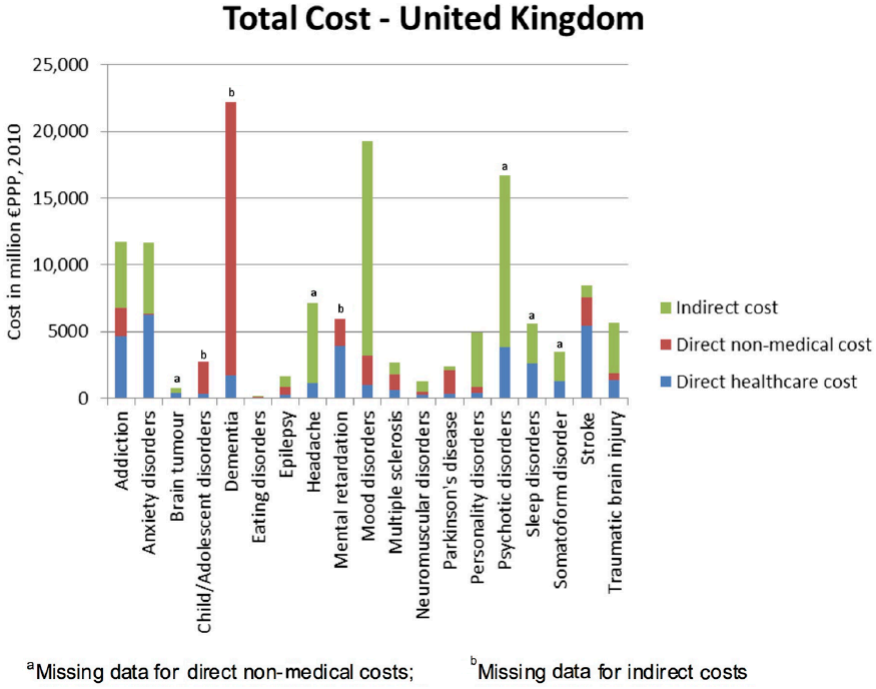
Total UK cost of individual brain disorders.

Taking account of the prevalence data, the total 2010 cost (in million € purchasing power parity (PPP)) of the individual disorders of the brain included in this study, ranked in order of magnitude, was as follows (Figure 3): dementia: €22,164; mood disorders: €19,238, psychotic disorders: €16,717; addiction: €11,719; anxiety disorders: €11,687; personality disorders:€4918; child/adolescent disorders: €2757; stroke: €8490; headache: €7119; mental retardation: €5975; traumatic brain injury: €5658; sleep disorders: €5630; somatoform disorder: €3514; multiple sclerosis: €2700; Parkinson’s disease: €2361; epilepsy: €1638; neuromuscular disorders: €1301; brain tumour: €766; eating disorders: €124. Thus, the five overall most costly disorders of the brain were dementia, mood disorder, psychotic disorder, anxiety disorder and addiction. The cost of these disorders was the highest despite having missing data on some cost types. It is noteworthy that, apart from psychosis, these most costly disorders ranked amongst those disorders with the lowest per-subject direct medical expenditure (<€3000 per subject; Figure 2).

## Discussion

Our report provides the most recent and comprehensive estimate of total costs for brain disorders, and brings together disorders that traditionally may have been separated into psychiatric and neurological specialties. We believe that a coordinated approach to disorders of the brain needs to be adopted, with comparable methodologies for both mental and neurological illness. This recognizes that many disorders (e.g. dementia, somatoform disorders, epilepsy) span both neurological and psychiatric domains, in healthcare, neuroscience and pharmaceutical research. The Department of Health (DOH) have also emphasized the need for “parity of esteem” between mental and physical health services (DOH, 2011).

The originating study (Gustavsson et al., 2011a) was based on the best currently available European data, applying a consistent methodology across a broad range of brain disorders. The model also enabled extrapolation to those disorders where no primary UK data could be found. We estimated the total annual cost of disorders of the brain in the UK in 2010 at approximately €134 billion. As expected, indirect costs associated with lost productivity constituted approximately 50% of the total burden, with direct healthcare and non-medical costs each comprising just over 25% of the budget (around €36 billion each). This contrasts with EU-wide data for brain disorders, in which direct costs constitute the majority (60%) of the costs (37% direct healthcare costs and 23% direct non-medical costs) (Gustavsson et al., 2011a), implying a relative deficit in UK investment in direct medical care.

The shortage of primary data for some disorders is an important source of uncertainty in these estimates (Gustavsson et al., 2011a) and may imply over, or under-estimates in some cases, including anxiety disorders and psychosis. In addition, the costs associated with early mortality were not included in the analysis. Although for some conditions these costs would be minimal, for example dementia, for others such as stroke, traumatic brain injury and tumours these productivity costs may be considerable, especially at the ‘cost per subject with the condition’ level. The exclusion of these costs also means that the results from this study may not be directly comparable with others that have included them, for example that of Luengo-Fernandez et al. (2012). Moreover, there are still remaining chronic and highly disabling disorders (e.g. body dysmorphic disorder) that could not be included in the analysis due to further limitations in the available data. Therefore, our estimate of the total cost of the disorders of the brain in the UK could be viewed as conservative. Indeed, our figure for the total annual UK cost associated with brain disorders (€134 billion) is broadly consistent with an earlier independent estimate from the DOH, which was itself largely based on extrapolation to the UK from global estimates (e.g. WHO, 2008), which estimated the total annual economic costs of mental illness (excluding neurological illness) in England alone to be £105 billion (DOH, 2011). However, it should be noted that this figure included an estimate for reduced quality of life, in addition to direct costs of services and lost productivity at work.

With regards annual direct expenditure, our estimates (€36 billion each for direct healthcare and non-medical services) are also compatible with available independent DOH estimates of direct expenditure. For example, in 2008/9, the NHS spent 10.8% of its annual secondary healthcare budget on mental illness alone (excluding neurological disorders), amounting to £10.4 billion (DOH, 2010a). Extended service costs for mental illness in England, which included NHS, social and informal care costs, amounted to the larger sum of £22.5 billion in 2007 (McCrone et al., 2008).

How then do the economic costs of disorders of the brain compare with those related to other costly somatic diseases? The DOH (2011) estimated mental ill health to be the single largest cause of disability in the UK, contributing up to 22.8% of the total burden, compared with 15.9% for cancer and 16.2% for cardiovascular disease. More recently, Luengo-Fernandez et al. (2012) estimated the relative 2007–8 UK healthcare and non-healthcare costs of dementia, stroke, cancer and coronary heart disease (CHD), representing the four leading causes of disability and mortality in Europe. The costs placed by dementia on the social care system alone (£9.3 billion) far outweighed the social care costs of cancer, CHD and stroke. Combining the annual costs of health and social care, dementia cost £10.5 billion, compared with £4.5 billion for cancer, £2.7 billion for stroke and £2.3 billion for CHD. After combining health and social care, informal care and productivity losses, dementia also had the highest annual cost at £23 billion, followed by cancer (£12 billion), CHD (£8 billion) and stroke (£5 billion). Yet, importantly, dementia was estimated to have the lowest direct healthcare costs (£1.2 billion), compared with £4.0 billion for cancer, £2.2 billion for CHD and £1.6 billion for stroke.

In this study, 2010 expenditure on direct medical and non-medical care of disorders of the brain was also low relative to the high indirect costs, especially for the common disorders of the brain for which the total cost to society is the highest (dementia, mood disorder, psychosis, anxiety disorder and addiction). However, it is precisely these same disorders for which UK translational neurosciences research has the realistic potential to produce transformative therapeutic advances (Insel et al., 2012), provided that public and commercial investment in research are prioritized. Moreover, despite potential constriction on health budgets, it is critically important that research, evaluation and innovation in mental disorders are recognized, in order to ‘learn what works and what does not’ and to disseminate this evidence into clinically effective and cost-effective practice (DOH, 2011).

According to UK government recommendations (Cooksey, 2006), health research priorities should be informed by an assessment of the impact of disease on the population and economy. However, according to a study of 2007–8 research expenditure (Luengo-Fernandez et al., 2012), by far the majority of health research funding in the UK has historically been directed towards cancer (total research expenditure = £590 million; 71%), and research spending on dementia (£50 million; 6%) and stroke (£23 million; 4%) has been comparatively seriously underfunded. In terms of economic burden, for every £1 million of health and social care costs attributable to each disease, cancer received £129,269 in research funding, CHD received £73,153, stroke received £8745 and dementia received just £4882. The reasons for these disparities are not well understood and likely to be complex. They may include ignorance of the magnitude of brain disorders, disproportionate fear of some diseases that leads to much greater charitable giving, an historical sense of tolerance or therapeutic nihilism for some disorders, and stigma over mental health problems.

The disparity in funding has recently been recognized and strategies have been initiated to address the NHS provision for stroke and dementia care based on translational research findings. For example, hyper-acute units are currently being established throughout England based on the finding that early thrombolysis can prevent completed stroke (DOH, 2010b). The 2012 ‘Prime Minister’s challenge on dementia’ (DOH, 2012) aims to double dementia research funding by 2015 and emphasizes both translational as well as basic neuroscience research, through the Medical Research Council, National Institute for Health Research and coordination of partnerships with industry. The Wellcome Trust Neurodegenerative Diseases Initiative additionally focuses multidisciplinary research on the causes, early diagnosis and therapeutic interventions for neurodegenerative diseases including Alzheimer’s disease, fronto-temporal dementia, Parkinson’s disease and motor neurone disease (http://www.wellcome.ac.uk/Funding/Biomedical-science/Funded-projects/Major-initiatives/Neurodegenerative-Diseases-Initiative/index.htm).

Looking to the future, we see that for the UK and for Europe as a whole (Gustavsson et al., 2011a), the health economic burden of disorders of the brain is likely to constitute the number one economic challenge for healthcare. The current estimates refer to 2010, but the costs over the next 20 years will not only stem from inflation, but also major increase in the disease burden associated with an ageing population. For example, the prevalence of dementia, associated with the highest total costs to the UK, is forecast to double over the next 30 years (Alzheimer’s Society, 2007).

With the total costs of disorders of the brain at €134 billion, and the additional prospect of an ageing population, it is clear that the UK must build upon the recent positive developments in the field of dementia and stroke and commit to a radical programme of research, innovation and investment for prevention and cost-effective treatment focusing on those brain disorders that are known to be the most costly for society, including dementia, mood disorder, psychosis, anxiety disorder and addiction. This not only calls for a review of government policy, but attention by all stakeholder groups, including industrial partners, patient representative organizations, educational establishments and the principal research funding bodies. To address the challenges and major economic threat posed by disorders of the brain, a transformation in knowledge and policy is required. To achieve this, it is necessary to develop a coordinated plan of action, at an EU and national level, to revitalize and transform the current scientific, healthcare and educational agenda. Running from 2014 to 2020, and with a dedicated budget of €24,598 million to strengthen top-level research in science aimed at improving EU productivity, Horizon 2020 – the EU’s new programme for research and innovation – could provide a pivotal role for enacting some of these changes.

## Recommendations

1. Given the cost of brain disorders, which are frequently chronic and relapsing, greater emphasis needs to be placed on prevention, early detection and early effective treatment.
2. There have been several initiatives in the UK and globally by governments and charities to address the underlying pathological processes, the translational models and new treatments for Alzheimer’s disease, for example the Prime Minister’s challenge, revised strategic priorities at the MRC, NIHR and the Wellcome Trust, as well as National research coordination via DenDRON and the Biomedical Research Centres in Dementia. Similar initiatives for the high-cost areas of mood disorders and psychotic disorders would greatly facilitate rapid advances in these areas. New developments at a European level, for example increasing the emphasis on brain disorders research under Horizon 2020, and in the NHS research directorate, for example networks such as the Mental Health Research Networks, should be encouraged and facilitated.
3. Screening for dementia, for example via the new NHS CQUIN screening of hospital admissions over 75 years of age, is increasingly common, though these NHS initiatives are sometimes transient and are yet to be proven to be effective. Given that mental health disorders disproportionately affect the young, there is a rationale for extending screening for common mental health disorders into adolescence and young adults in order to be able to treat more effectively and earlier.
4. Despite the prevalence of disorders of the brain, there is still severe stigma. Working with charities, patient advocacy groups and the media to reduce stigma will be important to facilitate patients seeking early treatment and thereby improving their functional outcome and wellbeing. This is particularly critical as some of these vulnerable patients are unable to advocate for themselves.
5. Private–public partnerships should be encouraged to promote novel drug development and to rapidly bring treatments to patients with brain disorders.
6. Efforts to remove hurdles to research must be supported at governmental level, such as removing inappropriate regulations within the European Clinical Trials Directive (2001/20/ec) or implementing faster and simpler processes for approval and governance of translational research.

## References

[bibr1-0269881113495118] AccessEconomics (2007) The cost of muscular dystrophy. Muscular Dystrophy Association. AccessEconomics.

[bibr2-0269881113495118] Alzheimer’s Society (2007) A report to the Alzheimer’s Society on the prevalence and economic cost of dementia in the UK produced by King’s College London and London School of Economics. London: Dementia UK.

[bibr3-0269881113495118] Andlin-Sobocki P Bengt Jönsson P Wittchen HU (2005) Cost of disorders of the brain in Europe. Eur J Neurol 12: 1–27. 15877774 10.1111/j.1468-1331.2005.01202.x

[bibr4-0269881113495118] APA (American Psychiatric Association) (1980) Diagnostic and statistical manual of mental disorders (3rd ed.). Washington, DC: American Psychiatric Association.

[bibr5-0269881113495118] APA (American Psychiatric Association) (1987) Diagnostic and statistical manual of mental disorders (3rd ed., revised). Washington, DC: American Psychiatric Association.

[bibr6-0269881113495118] APA (American Psychiatric Association) (1994) Diagnostic and statistical manual of mental health disorders (4th ed). Washington DC: American Psychiatric Association.

[bibr7-0269881113495118] Balakrishnan R Allender S Scarborough P (2009) The burden of alcohol-related ill health in the United Kingdom. J Public Health (Oxf) 31: 366–373. 19493915 10.1093/pubmed/fdp051

[bibr8-0269881113495118] Bebbington PE Angermeyer M Azorin JM (2005) The European Schizophrenia Cohort (EuroSC): A naturalistic prognostic and economic study. Soc Psychiatry Psychiatr Epidemiol 40: 707–717. 16151597 10.1007/s00127-005-0955-5

[bibr9-0269881113495118] Blomqvist P Lycke J Strang P (2000) Brain tumours in Sweden 1996: Care and costs. J Neurol Neurosurg Psychiatry 69: 792–798. 11080235 10.1136/jnnp.69.6.792 PMC1737161

[bibr10-0269881113495118] Cockerell OC Hart YM Sander JW (1994) The cost of epilepsy in the United Kingdom: An estimation based on the results of two population-based studies. Epilepsy Res 18: 249–260. 7805646 10.1016/0920-1211(94)90045-0

[bibr11-0269881113495118] Collins PY Patel V Joestl SS (2011) Grand challenges in global mental health. Nature 475 (7354): 27–30. 21734685 10.1038/475027a PMC3173804

[bibr12-0269881113495118] Cooksey D (2006) A Review of UK Health Research Funding. London: HMSO. 10.1136/bmj.39059.444120.80 PMC1702444 17170394

[bibr13-0269881113495118] Das GR Guest JF (2002) Annual cost of bipolar disorder to U.K. society. Br J Psychiatry 180: 227–233. 11872515 10.1192/bjp.180.3.227

[bibr14-0269881113495118] DOH (Department of Health) (2010a) Programme budgeting tools and data. National expenditure data. Available at http://webarchive.nationalarchives.gov.uk/+/www.dh.gov.uk/en/Managingyourorganisation/Financeandplanning/programmebudgeting/dh_075743#_3 (accessed 11 July 2013).

[bibr15-0269881113495118] DOH (Department of Health) (2010b) Progress in improving stroke care. In: Report by the comptroller and auditor general. London: The Stationery Office Available at: http://www.nao.org.uk/wp-content/uploads/2010/02/0910291.pdf. (accessed 11 July 2013).

[bibr16-0269881113495118] DOH (Department of Health) (2011) No health without mental health: A cross-Government mental health outcomes strategy for people of all ages. Available at: https://www.gov.uk/government/uploads/system/uploads/attachment_data/file/135434/dh_123990.pdf.pdf (accessed 12 July 2013).

[bibr17-0269881113495118] DOH (Department of Health) (2012) Prime Minister’s challenge on dementia – delivering major improvements in dementia care and research by 2015. London: Williams Lea Available at: https://www.gov.uk/government/uploads/system/uploads/attachment_data/file/200030/9535-TSO-2900951-PM_Challenge_Dementia_ACCESSIBLE.PDF (accessed 12 July 2013).

[bibr18-0269881113495118] Espèrou H Jars-Guincestre MC Bolgert F (2000) Cost analysis of plasma-exchange therapy for the treatment of Guillain–Barre syndrome. French Cooperative Group on plasma exchange in Guillain–Barre syndrome. Intensive Care Med 26: 1094–1100. 11030166 10.1007/s001340051323

[bibr19-0269881113495118] Godet-Cayré V Pelletier-Fleury N Le VM (2006) Insomnia and absenteeism at work. Who pays the cost? Sleep 29: 179–184. 16494085 10.1093/sleep/29.2.179

[bibr20-0269881113495118] Godfrey C Stewart D Gossop M (2004) Economic analysis of costs and consequences of the treatment of drug misuse: 2-year outcome data from the National Treatment Outcome Research Study (NTORS). Addiction 99: 697–707. 15139868 10.1111/j.1360-0443.2004.00752.x

[bibr21-0269881113495118] Gustavsson A Svensson M Jacobi F (2011a) Cost of disorders of the brain in Europe 2010. Eur Neuropsychopharmacol 21: 718–779. 21924589 10.1016/j.euroneuro.2011.08.008

[bibr22-0269881113495118] Gustavsson A Brinck P Bergvall N (2011b) Predictors of costs of care in Alzheimer’s disease: A multinational sample of 1222 patients. Alzheimers Dement 7: 318–327. 21575872 10.1016/j.jalz.2010.09.001

[bibr23-0269881113495118] Hakkaart-van RL Zwirs BW Bouwmans C (2007) Societal costs and quality of life of children suffering from attention deficient hyperactivity disorder (ADHD). Eur Child Adolesc Psychiatry 16: 316–326. 17483870 10.1007/s00787-007-0603-6

[bibr24-0269881113495118] Heider D Bernert S Konig HH (2009). Direct medical mental health care costs of schizophrenia in France, Germany and the United Kingdom — findings from the European Schizophrenia Cohort (EuroSC). Eur Psychiatry 24: 216–224. 19328658 10.1016/j.eurpsy.2008.12.013

[bibr25-0269881113495118] Insel TR Sahakian BJ Voon V A plan for mental health. Nature 483, 269 (2012). Available at: http://dx.doi.org/10.1038/483269a 10.1038/483269a 22422245

[bibr26-0269881113495118] Jacoby A Buck D Baker G (1998) Uptake and costs of care for epilepsy: Findings from a U.K. regional study. Epilepsia 39: 776–786. 9670907 10.1111/j.1528-1157.1998.tb01164.x

[bibr27-0269881113495118] Jacobi F Wittchen HU Holting C (2002) Estimating the prevalence of mental and somatic disorders in the community: Aims and methods of the German National Health Interview and Examinations Survey. Int J Method Psychiatr Res 11: 1–18. 10.1002/mpr.118 PMC6878553 12459800

[bibr28-0269881113495118] Jennum P Riha RL (2009) Epidemiology of sleep apnoea/hypopnoea syndrome and sleep-disordered breathing. Eur Respir J 33: 907–914. 19336593 10.1183/09031936.00180108

[bibr29-0269881113495118] Jennum P Kjellberg J (2010) The socio-economical burden of hypersomnia. Acta Neurol Scand 121: 265–270 20047573 10.1111/j.1600-0404.2009.01227.x

[bibr30-0269881113495118] Jennum P Kjellberg J (2011). Health, social and economical consequences of sleep-disordered breathing: A controlled national study. Thorax 66: 560–566. 21199816 10.1136/thx.2010.143958

[bibr31-0269881113495118] Kessler RC Aguilar-Gaxiola S Alonso J (2009) The WHO World Mental Health (WMH) Surveys. Psychiatrie (Stuttg) 6: 5–9. 21132091 PMC2995950

[bibr32-0269881113495118] Klin A Lemish D (2008) Mental disorders stigma in the media: Review of studies on production, content, and influences. J Health Commun 13: 434–449. 18661386 10.1080/10810730802198813

[bibr33-0269881113495118] Knapp M Romeo R Beecham J (2009) Economic cost of autism in the UK. Autism 13: 317–336. 19369391 10.1177/1362361309104246

[bibr34-0269881113495118] Kobelt G Berg J Lindgren P (2006) Costs and quality of life of multiple sclerosis in the United Kingdom. Eur J Health Econ 7(Suppl. 2): 96–104. 10.1007/s10198-006-0380-z 17310341

[bibr35-0269881113495118] Krauth C Buser K Vogel H (2002) How high are the costs of eating disorders – anorexia nervosa and bulimia nervosa – for German society? Eur J Health Econ 3: 244–250. 15609150 10.1007/s10198-002-0137-2

[bibr36-0269881113495118] Leger D Levy E Paillard M (1999) The direct costs of insomnia in France. Sleep 22(Suppl. 2): 394–401. 10394613

[bibr37-0269881113495118] Linde M Gustavsson A Stovner LJ (2012) The cost of headache disorders in Europe: The Eurolight project. Eur J Neurol 19: 703–711. 22136117 10.1111/j.1468-1331.2011.03612.x

[bibr38-0269881113495118] Luengo-Fernandez R Gray AM Rothwell PM (2009) Costs of stroke using patient-level data: A critical review of the literature. Stroke 40: 18–23. 19109540 10.1161/STROKEAHA.108.529776

[bibr39-0269881113495118] Luengo-Fernandez R Leal J Gray AM (2012) UK research expenditure on dementia, heart disease, stroke and cancer: are levels of spending related to disease burden? Eur J Neurol 19: 149–154. 21951976 10.1111/j.1468-1331.2011.03500.x

[bibr40-0269881113495118] Mahdi-Rogers M Kazmi M Ferner R (2009) Autologous peripheral blood stem cell transplantation for chronic acquired demyelinating neuropathy. J Peripher Nerv Syst 14: 118–124. 19691534 10.1111/j.1529-8027.2009.00221.x

[bibr41-0269881113495118] McCrone P Dhanasiri S Patel A (2008) Paying the price: the cost of mental health care in England to 2026. London: King’s Fund Available at: http://www.kingsfund.org.uk/sites/files/kf/Paying-the-Price-the-cost-of-mental-health-care-England-2026-McCrone-Dhanasiri-Patel-Knapp-Lawton-Smith-Kings-Fund-May-2008_0.pdf (accessed 11 July 2013).

[bibr42-0269881113495118] McCrone P Allcock LM Burn DJ (2007) Predicting the cost of Parkinson’s disease. Mov Disord 22: 804–812. 17290462 10.1002/mds.21360

[bibr43-0269881113495118] Morris S Ridley S Lecky FE (2008) Determinants of hospital costs associated with traumatic brain injury in England and Wales. Anaesthesia 63: 499–508. 18412648 10.1111/j.1365-2044.2007.05432.x

[bibr44-0269881113495118] Murray CJL Lopez AD (1996) The global burden of disease: A comprehensive assessment of mortality and disability from diseases, injuries and risk factors in 1990 and projected to 2020. Global Burden of disease and Injury Series. Cambridge: Harvard University Press.

[bibr45-0269881113495118] Murray CJL Lopez AD (1997) Alternative projections of mortality and disability by cause 1990–2020: Global Burden of Disease Study. Lancet 349: 1498–1504. 9167458 10.1016/S0140-6736(96)07492-2

[bibr46-0269881113495118] Nutt D Goodwin G (2011) ECNP Summit on the future of CNS drug research in Europe 2011: Report prepared for ECNP by David Nutt and Guy Goodwin. Eur Neuropsychopharmacol 21: 495–499. 21684455 10.1016/j.euroneuro.2011.05.004

[bibr47-0269881113495118] Polder JJ Meerding WJ Bonneux L (2002) Healthcare costs of intellectual disability in the Netherlands: A cost-of-illness perspective. J Intellect Disabil Res 46: 168–178. 11869388 10.1046/j.1365-2788.2002.00384.x

[bibr48-0269881113495118] Polinder S Meerding WJ van Baar ME (2005) Cost estimation of injury-related hospital admissions in 10 European countries. J Trauma 59: 1283–1290. 16394898 10.1097/01.ta.0000195998.11304.5b

[bibr49-0269881113495118] Priebe S Matanov A Jankovic GJ (2009) Consequences of untreated posttraumatic stress disorder following war in former Yugoslavia: Morbidity, subjective quality of life and care costs. Croat M J 50: 465–475. 19839070 10.3325/cmj.2009.50.465 PMC2765735

[bibr50-0269881113495118] Priebe S Gavrilovic JJ Matanov A (2010) Treatment outcomes and costs at specialized centers for the treatment of PTSD after the war in former Yugoslavia. Psychiatr Serv 61: 598–604. 20513683 10.1176/ps.2010.61.6.598

[bibr51-0269881113495118] Prince M Patel V Saxena S (2007) No health without mental health. Lancet 370: 859–877. 17804063 10.1016/S0140-6736(07)61238-0

[bibr52-0269881113495118] Romeo R Knapp M Scott S (2006). Economic cost of severe antisocial behaviour in children—and who pays it. Br J Psychiatry 188: 547–553. 16738345 10.1192/bjp.bp.104.007625

[bibr53-0269881113495118] Schepelmann K Winter Y Spottke AE (2010) Socioeconomic burden of amyotrophic lateral sclerosis, myasthenia gravis and facioscapulohumeral muscular dystrophy. J Neurol 257: 15–23. 19629566 10.1007/s00415-009-5256-6

[bibr54-0269881113495118] Soeteman DI Hakkaart-van RL Verheul R (2008) The economic burden of personality disorders in mental health care. J Clin Psychiatry 69: 259–265. 18363454 10.4088/jcp.v69n0212

[bibr55-0269881113495118] Swingler RJ Davidson DL Roberts RC (1994) The cost of epilepsy in patients attending a specialist epilepsy service. Seizure 3: 115–120. 8081637 10.1016/s1059-1311(05)80201-8

[bibr56-0269881113495118] Thomas CM Morris S (2003) Cost of depression among adults in England in 2000. Br J Psychiatry 183: 514–519. 14645022 10.1192/bjp.183.6.514

[bibr57-0269881113495118] Van Asselt AD Dirksen CD Arntz A (2007) The cost of borderline personality disorder: Societal cost of illness in BPD-patients. Eur Psychiatry 22: 354–361. 17544636 10.1016/j.eurpsy.2007.04.001

[bibr58-0269881113495118] Van Hout B Gagnon D Souetre E (1997) Relationship between seizure frequency and costs and quality of life of outpatients with partial epilepsy in France, Germany, and the United Kingdom. Epilepsia 38: 1221–1226. 9579924 10.1111/j.1528-1157.1997.tb01220.x

[bibr59-0269881113495118] Wasserfallen JB Ostermann S Leyvraz S (2005) Cost of temozolomide therapy and global care for recurrent malignant gliomas followed until death. Neuro Oncol 7: 189–195. 15831237 10.1215/S1152851704000687 PMC1871888

[bibr60-0269881113495118] Wittchen HU Jacobi F (2005) Size and burden of mental disorders in Europe – a critical review and appraisal of 27 studies. Eur Neuropsychopharmacol 15: 357–376. 15961293 10.1016/j.euroneuro.2005.04.012

[bibr61-0269881113495118] Wittchen HU Jacobi F Rehm J (2011) The size and burden of mental disorders and other disorders of the brain in Europe 2010. Eur Neuropsychopharmacol 21: 655–679. 21896369 10.1016/j.euroneuro.2011.07.018

[bibr62-0269881113495118] Wolstenholme J Fenn P Gray A (2002) Estimating the relationship between disease progression and cost of care in dementia. Br J Psychiatry 181: 36–42. 12091261 10.1192/bjp.181.1.36

[bibr63-0269881113495118] WHO (World Health Organization) (1993) The ICD-10 classification of mental and behavioural disorders: Diagnostic criteria for research. World Health Organization.

[bibr64-0269881113495118] WHO (World Health Organization) (2002) The World Health Report 2002 – reducing risks, promoting healthy life. Available at: http://www.who.int/whr/2002/en/whr02_en.pdf (accessed 12 July 2013).

[bibr65-0269881113495118] WHO (World Health Organization) (2008) The Global burden of disease 2004 Update. Available at: http://www.who.int/healthinfo/global_burden_disease/GBD_report_2004update_full.pdf (accessed 11 July 2011).

